# In vivo analysis of GenePro, a lentivral therapeutic vaccine

**DOI:** 10.1186/1471-2334-14-S2-P2

**Published:** 2014-05-23

**Authors:** Mariel Selbovitz, David Miller

**Affiliations:** 1 Cornell AIDS Clinical Trials Group, New York, USA

## Introduction

Despite advances in HIV commercial therapies, including fixed-dose combinations, unprecedented proportions of HIV patients remain without therapeutic options due to lack of access to treatment and epidemiologically significant proportions of multi-class drug resistance that are driving morbidity levels in both developed and resource-poor settings. The need to emphasize the development of therapeutic options with the potential to abrogate these correlating trends are receiving insufficient dedication of resources, as new clinical strategies do not address these enduring disparities. GenePro, a therapeutic vaccine candidate, has shown preclinical efficacy via publicly sponsored research that demonstrates the potential to address a current therapeutic issue.

## Materials and methods

The GenePro therapeutic construct includes strong lentiviral promoter and 7 out of 9 HIV genes: Gag, pol, tat, rev, vpu, env and nef. In a preclinical study, kinetic analyses on splenocytes of BALB/c mice that were immunized by a single injection with a GenePro were performed. IFN-gamma-ELISPOT and multiparametric FACS analysis were conducted to characterize the induced CMI response. Animals immunized with a single high dose of GenePro were monitored longitudinally for vaccine-induced IR using multiparametric flow cytometry-based assays.

## Results

Comprehensive analysis demonstrated the capacity of a single high dose of HIV DNA vaccine alone to induce long-lasting and polyfunctional T-cell responses in the nonhuman primate model. In preclinical studies, GenePro has been found to generate the HIV-specific memory cells found in elite controllers and reconstitution of the immune system. The TriGrid delivery system increases DNA delivery efficiency by up to 1,000 fold. This approach has less toxicity and no drug resistance and may allow for STIs from ART.

## Conclusion

Data from initial investigations of GenePro indicate improvements in immune response and generation of HIV-specific memory cells that induced immune responses by altered HIV DNA constructs, which synthetically mimic virologic responses of elite controllers. Advancement of GenePro into human studies will serve as a hallmark in continuing commercial efforts to discover a universal address for the global AIDS crisis.

